# Supplementary material for: Patterns of Intron Gain and Loss in Fungi
Source: PLoS Biol. 2004 Nov 30;2(12):e422. doi: 10.1371/journal.pbio.0020422 (PMC532390; doi:10.1371/journal.pbio.0020422)
Supplement: Table S1 — Also available at http://genes.mit.edu/NielsenEtAl/. (4.3 MB ZIP). [file pbio.0020422.st001.zip › NielsenEtAl/html/965.html]

AN4232.1.NCU04503.1.MG00087.1.FG04027.1


```
 CLUSTAL W (1.82) Multiple Sequence Alignments - Introns Inserted


Sequence 1: NCU04503.1	482 aa
Sequence 2: FG04027.1	454 aa
Sequence 3: MG00087.1	474 aa
Sequence 4: AN4232.1	442 aa
Alignment Length: 490 aa
Number Identitical Residues: 193 aa
Alignment Score (without introns) 10227


MG00087.1 	MARSRAKRRTHAGAKNPNQQKGPINTGNASIRDPKSMVIRIGAGEVGSSVSQLATDVRRV
NCU04503.1	MARKRVKKRTHVGAKNPVSSAALN--GHADRKDPKSMVIRIGAGEVGTSISQLATDVRRV
FG04027.1 	MARKRTKKRTHLGASNPDVDSA----GHASAKDPKSMVIRIGAGEVGSSVSQLAADVRKV
AN4232.1  	MAKH---TKSHAHPKPPSGAAKGKGSAASVSKTPKSMVIRIGGSRVGSSVSQLVKDVRLM
          	**:     ::*  .. *       .:. :  : *********...**:*:***. *** :

MG00087.1 	MEPGTASRLK~ERRANKLRDYLVMCGPLGVTHLLLFSRSESGNVNLRVALTPRGPTLHFR
NCU04503.1	MEPGTATRLK~ERKANRLRDYVTMCGPLGVTHLLLFSRSESGNTNLRLAIAPRGPTFNFR
FG04027.1 	MEPGTASRLK~ERRGNRLKDYAVMCGPLGVTHLMLFSRSETGNTNLRMALTPRGPTLNFR
AN4232.1  	MEPDTAVRLK0ERKSNRLRDYAVMAGPLGVTHFLLFSKSSTGNTNMRLALTPRGPTLNFK
          	***.** *** **:.*:*:** .*.*******::***:*.:**.*:*:*::*****::*:

MG00087.1 	VEKYSLTKDVQKSQRRPKGQGKEFVTSPL0LVMNNFSSSSADAPSKTPKHLESLTTTVFQ
NCU04503.1	VDKYSLAKDVRRAQRHPKGGGKEFLTPPL0LVMNNFTDPNADANSKVPKHLESITTTAFQ
FG04027.1 	VEKYSLCKDVQRAQKHPKGGGKEFITPPL0LVMNNFTRPDSDNKSKVPRHLESLATTVFQ
AN4232.1  	VESYSLCRDVEKALKRPRGGGQDHKTPPL~LVMNNFNSPNADENSKVPKRLESLTTTVFQ
          	*:.*** :**.:: ::*:* *::. *.** ******. ..:*  **.*::***::**.**

MG00087.1 	SLFAPINPQKTSLKSIRRVLLMNREQSKEMD--GSFIIDFRHYAITTKPTGISKPLKRLN
NCU04503.1	SLFPPINPQRTPLKSIRRVLLLNREKSPEND--GSFIINFRHYAITTKAVGMSKPLRRLN
FG04027.1 	SLFPPINPQATPLKSIRRVLLLNRETSEEDD--GTFIVNFRHYAITTKSTTVSKPLRRLN
AN4232.1  	SLFPPINPQATPLKSIRRVMLLNREPASESDKEGSYILNLRHYAITTRKTGIPKRIRRLD
          	***.***** *.*******:*:*** : * *..*::*:::*******: . :.* ::**:

MG00087.1 	AAEKLLSGKTGKKGGLPNLNKLQDIADYMIGGEDGSGYMTDGGTSGSEAETDAEVEVLEP
NCU04503.1	AAEKLIKSKS-RKGGLPNLGKLRDISEFMIGGEDGQGYMTDG-TSGSEYDTDAEIEILDQ
FG04027.1 	AAEQFVTSKTNRKGKMPNLGKLEDVADFLIGGEAGDGYMTDA-TSGSEMDTDAEVEILDS
AN4232.1  	PKE--VRNREKHKSAVPNLGKLEDAADYLLDP-SAAGYTSAS---ETELDTDAEVEVAGT
          	. *  : .:  :*. :***.**.* :::::.   . ** : .    :* :****:*:   

MG00087.1 	TSRKVLNAKARAAMREAENGDEGSQAGTEQQNVEKRAVKLAELGPRMRLRLLKVEEGVCA
NCU04503.1	GPKKVKSAKARAAIAAVEAAEEEEEG--HDDNVERRAVKLVELGPRMRLRMTKVEEGMCS
FG04027.1 	TTRKVLSAKARQAAAQNETEPEAEE-----ENVERRAVKLVELGPRMRLRLTKVEEGLAS
AN4232.1  	TTRKVLTKREMQRMKTGEKVEKKTTT----AEVEKRAVKLVELGPRMRLRLIKVEEGLCE
          	 .:** . :        *   :         :**:*****.*********: *****:. 

MG00087.1 	GKVMWHEYINKSKEEIKAMEKKWEQKQREKEARKKVQKENVERKRKEKEEQKKNSKKSGE
NCU04503.1	GKVLWHEYVHKTQEEIRELEKKWEQKRREKEARKKQQKANVEKKKAEKAAHKKATGGKGK
FG04027.1 	GKVMWHEYIHKNKDEIKELEKRWEQRRQEKDARKKEQKANVERKKAAKTKD----GNKGE
AN4232.1  	GRVMWHDYIHKSQQEVDALDKTWDQKIKEKEARKKLQRENVERKKQEKAKAR----AEGK
          	*:*:**:*::*.::*:  ::* *:*: :**:**** *: ***:*:  *         .*:

MG00087.1 	NADGDDD----DEDEMDVDYDSDDYEFDSEGMAGDAETLVNEKAEEDGEWEDEEEEIAAG
NCU04503.1	DDSDDEDEMDMDDYPYDSDMYDAEDGFDSEGLAGDAEEQANTKMEQDGEWEDEEEEIAQE
FG04027.1 	DDDEE------DEFEYDSDMD--VDEFDSEGLAGDAEFKVNEKMEEDGEWEDEEEEIANS
AN4232.1  	EVEDD-------EDDEDVDMD---DEWIS-----DDEEEQNQEVDEEDEGDESIDE----
          	: . :       :   * *       : *     * *   * : :::.* ::. :*    

MG00087.1 	AG----------
NCU04503.1	AQAKGKKKPALK
FG04027.1 	------------
AN4232.1  	------------
          	
```
